# Background-free *in-vivo* Imaging of Vitamin C using Time-gateable Responsive Probe

**DOI:** 10.1038/srep14194

**Published:** 2015-09-16

**Authors:** Bo Song, Zhiqing Ye, Yajie Yang, Hua Ma, Xianlin Zheng, Dayong Jin, Jingli Yuan

**Affiliations:** 1 State Key Laboratory of Fine Chemicals, School of Chemistry,Dalian University of Technology, Dalian 116024, P. R. China; 2 Advanced Cytometry Labs, ARC Centre of Excellence for Nanoscale BioPhotonics (CNBP),Macquarie University, NSW 2109, Sydney, Australia

## Abstract

Sensitive optical imaging of active biomolecules in the living organism requires both a molecular probe specifically responsive to the target and a high-contrast approach to remove the background interference from autofluorescence and light scatterings. Here, a responsive probe for ascorbic acid (vitamin C) has been developed by conjugating two nitroxide radicals with a long-lived luminescent europium complex. The nitroxide radical withholds the probe on its “off” state (barely luminescent), until the presence of vitamin C will switch on the probe by forming its hydroxylamine derivative. The probe showed a linear response to vitamin C concentration with a detection limit of 9.1 nM, two orders of magnitude lower than that achieved using electrochemical methods. Time-gated luminescence microscopy (TGLM) method has further enabled real-time, specific and background-free monitoring of cellular uptake or endogenous production of vitamin C, and mapping of vitamin C in living *Daphnia magna*. This work suggests a rational design of lanthanide complexes for background-free small animal imaging of biologically functional molecules.

Ascorbic acid (vitamin C), an essential nutrient for humans, has been well known for a long time because of its essential role in the prevention of deficiency diseases, for example, scurvy^1^. It helps also in healthy cell development, calcium absorption, the healing of injuries and burns, and the synthesis of collagen, blood vessels, cartilage, bone and tendons^2^,^3^,^4^. In recent years vitamin C has attracted considerable attentions for its role in the promotion of healthy brain aging and potential in cancer treatment^5^,^6^. An increasing number of studies have been focused on clarifying the biological roles of vitamin C, but one of the most important difficulties preventing these studies attributes to the lacking of a robust method to monitor vitamin C in living systems.

The most commonly reported analytical methods for vitamin C are based on electrochemical approaches^7^,^8^, but the interference from other non-specific molecules, such as uric acid and dopamine, can seriously disturb the results in real-time monitoring of vitamin C in living samples. The fluorescent microscopy using a responsive fluorescent probe could provide superior advantages in high sensitivity and selectivity to investigate vitamin-induced inhibition of cancer cell growth, but developing such functional probes is still in its early stage^9^. A vitamin C-responsive fluorescence probe can be designed by linking a nitroxide radical to a fluorophores^9^,^10^,^11^, in which design the nitroxide radical moiety not only quenches the fluorescence of the probe by intramolecular electron exchange interaction but also specifically reacts with vitamin C to switch on the fluorescence of the probe^9^,^10^,^11^. But due to their insufficient sensitivity, this kind probe can only provide quantification of vitamin C in aqueous media at relatively high concentration range (>μM)^10^,^11^, and also requires long incubation time and high concentration of extracellular vitamin C for the intracellular imaging demonstration^9^.

On the other hand, time-gated detection technique using lanthanide complexes as luminescence probes has been widely used to achieve highly sensitive luminescence immunoassay^12^, DNA hybridization assay^13^, evaluation of enzyme activities^14^ and detection of various intracellular analytes^15^,^16^,^17^,^18^,^19^,^20^. Compared to the conventional organic fluorescence probes, lanthanide-based probes exhibit several unique features including large Stokes shifts, sharp emission profiles and long emission lifetimes. The exceptionally long luminescence lifetime (>10 μs) enables time-gated luminescence microscopy (TGLM) imaging of rare cell types^21^ and trace amount of molecules^22^ with high signal-to-noise contrast ratio and therefore ultra-high sensitivity, since all the short-lived autofluorescence from biological components and excitation scatterings by the optics can be efficiently eliminated (time-gated). Thus, once a lanthanide complex-based responsive probe can be developed specific for vitamin C TGLM imaging, it will be applied to monitor vitamin C’s distribution and dynamic activities in living cells and laboratory animals.

In this work, by linking a stable free radical of 2,2,6,6-tetramethyl-1-oxy-4-aminopiperidine (4-amino-TEMPO) to a highly luminescent Eu^3+^ complex, 1,2-bis[4′-(1′′,1′′,1′′,2′′,2′′,3′′,3′′-heptafluoro-4′′,6′′-hexanedion-6′′-yl)-benzyl]-4-chlorosulfobenzene-Eu^3+^ (BHHBCB-Eu^3+^), we designed and synthesized a time-gated luminescence probe for detecting vitamin C. The designed Eu^3+^ complex probe, TOB-Eu^3+^ (Fig. 1), is weakly luminescent due to the strong luminescence quenching effect of two TEMPO radicals. It can specifically react with vitamin C to afford its hydroxylamine derivative, TOHB-Eu^3+^, accompanied by a remarkable luminescence enhancement, which allows the probe to be favorably used for the *in vivo* time-gated luminescence detection of vitamin C in aqueous media, living cell samples and laboratory animals.

## Results

### The synthesis

The new ligand TOB was synthesized by covalently conjugating 4-amino-TEMPO to BHHBCB through the formation of sulfonamide. The corresponding complex, TOB-Eu^3+^, was synthesized by mixing double molar of TOB with europium(III) triflate in acetonitrile, and the reduced product, TOHB-Eu^3+^, was obtained by reacting TOB-Eu^3+^ with vitamin C in acetonitrile. All the above compounds were well characterized by HPLC, ESI-MS, and CHN elementary analyses (Figure S1–S2).

### Luminescence properties

**Figure 2:**
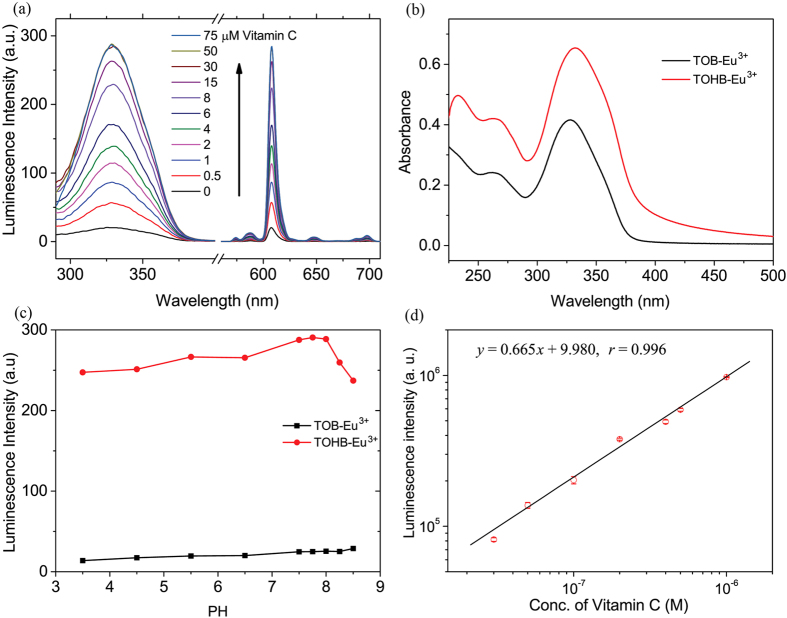
Basic characterizations of the responsive probe TOB-Eu^3+^ and its on-state compound TOHB-Eu^3+^. (**a**) Time-gated excitation (λ_em _= 608 nm) and emission (λ_ex _= 330 nm) spectra of TOB-Eu^3+^ (0.5 μM) in the presence of different concentrations of vitamin C in 1:1 ethanol-0.05 M Tris-HCl buffer of pH 7.5. (**b**) UV-vis absorption spectra of TOB-Eu^3+^ (10 μM, black) and TOHB-Eu^3+^ (10 μM, red) in 1:1 ethanol-0.05 M Tris-HCl buffer of pH 7.5. (**c**) Effects of pH on the luminescence intensities of TOB-Eu^3+^ (0.5 μM, black) and TOHB-Eu^3+^ (0.5 μM, red) in solutions of 0.05 M Tris-HCl/ethanol (v/v = 1:1) with different pH values. (**d**) Calibration curve for vitamin C measured on Perkin Elmer Victor 1420 multilabel counter.

The luminescence properties of TOB-Eu^3+^ and TOHB-Eu^3+^ were determined in 1:1 ethanol-0.05 M Tris-HCl buffer of pH 7.5, and the results were summarized in Table 1. Both TOB-Eu^3+^ and TOHB-Eu^3+^ showed almost the same maximum excitation and emission wavelengths at ~330 nm and 608 nm, respectively, and exhibited a typical Eu^3+^ emission pattern (Fig. 2a) consisting of several discrete bands between 580 and 710 nm corresponding to the ^5^D_0_→^7^F_J_ (J = 0–4) transitions of Eu^3+^. There is no remarkable differences in the UV spectrum profiles between TOB-Eu^3+^ and TOHB-Eu^3+^, but the molar absorption coefficient of TOHB-Eu^3+^ increased by 0.5 times than that of TOB-Eu^3+^ (Fig. 2b). After the vitamin C reduction, the luminescence quantum yield and lifetime of TOB-Eu^3+^ were 10-fold and 2-fold increased, respectively. These results suggest that TOB-Eu^3+^ can act as a turn-on luminescence probe for the time-gated luminescence detection of vitamin C.

### pH stability

Furthermore, the effects of pH on the luminescence intensities of TOB-Eu^3+^ and TOHB-Eu^3+^ were examined. As shown in Fig. 2c, the luminescence intensity of TOB-Eu^3+^ is stable in the range of pH 3.5–8.5, while that of TOHB-Eu^3+^ shows slight increases when pH is increased from 3.5 to 8.0. At pH > 8.0, luminescence intensity of TOHB-Eu^3+^ shows distinct decreases, which might be due to the deprotonation of HO-TEMPO moiety in TOHB-Eu^3+^.

### Quantitative detection

To evaluate the applicability of TOB-Eu^3+^ as a time-gated luminescence probe for the quantitative detection of vitamin C, the time-gated emission spectra of TOB-Eu^3+^ in the presence of different concentrations of vitamin C were recorded in 1:1 ethanol-0.05 M Tris-HCl buffer of pH 7.5. As shown in Fig. 2a, upon reaction with different concentrations of vitamin C, the luminescence intensity of the probe gradually increased with up to 14.5-fold enhancement, which indicates the formation of TOHB-Eu^3+^ as the turned-on state of the probe with strong luminescence. This exhibits much higher contract ratio than that achieved by the reported phthalocyaninatosilicon probe^9^. When the measurements were carried out on a more sensitive Perkin Elmer Victor 1420 multilabel counter (96-well microtiter plates as cuvettes) in time-gated detection mode, the dose-dependent luminescence enhancement showed a good linearity between the luminescence intensity and the vitamin C concentration (Fig. 2d). The detection limit for vitamin C, calculated as the concentration corresponding to triple standard deviations of the background signal, is 9.1 nM, achieving two orders of magnitude lower than that of the electrochemical methods^23^.

### Specificity

**Figure 3:**
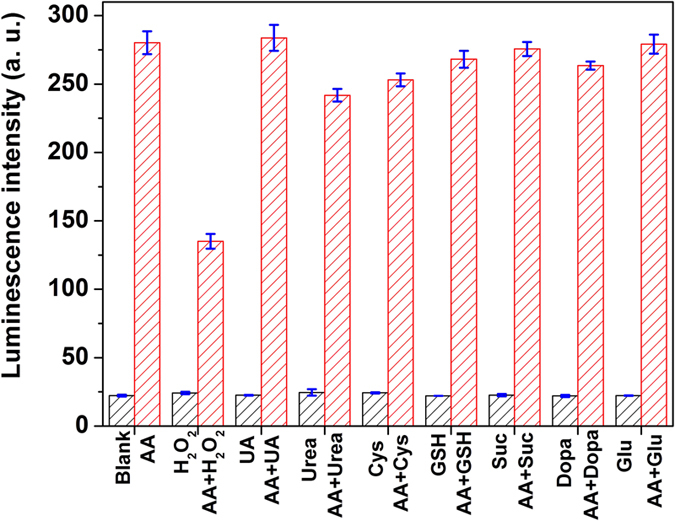
Specificity evaluations. Luminescence intensities of the products of TOB-Eu^3+^ (5.0 μM) reacted with 200 μM different biological reductants and 400 μM H_2_O_2_ in 1:1 ethanol-0.05 MTris-HCl buffer of pH 7.5 (black shade bars). The red shade bars show the luminescence intensities of the products of TOB-Eu^3+^ (5.0 μM) reacted with 200 μM different biological reductants and 400 μM H_2_O_2_ in the presence of ascorbic acid (AA) (100 μM) in the buffer.

To evaluate the response specificity of TOB-Eu^3+^ to vitamin C, the reactions of TOB-Eu^3+^ with different biological reductants were examined under the same conditions. As shown in Fig. 3, the luminescence intensity of TOB-Eu^3+^ did not respond to reduced glutathione (GSH), dopamine (Dopa), cysteine (Cys), succinimide (Suc), uric acid (UA), urea, glucose (Glu) and H_2_O_2_, whereas a remarkable increase was observed after the probe reacted with vitamin C (ascorbic acid, simplified as AA in Fig. 3). Furthermore, the competition experiments were carried out to evaluate the luminescence response of TOB-Eu^3+^ to vitamin C in the presence of coexisting biological reductants. The results indicate that, except for H_2_O_2_, the chosen biological reductants have no significant influences on the luminescence response of TOB-Eu^3+^ to vitamin C (Fig. 3, red shade bars). The effect of H_2_O_2_ can be attributed to the oxidation of vitamin C by H_2_O_2_, which forms dehydroascorbic acid (DHA) to block the reaction of TOB-Eu^3+^ with vitamin C, and thus the luminescence response of TOB-Eu^3+^ to vitamin C is obviously reduced. It was reported that vitamin C at high levels can act as prodrug to deliver a significant flux of H_2_O_2_ to tumors^24^, so the mentioned finding could be useful for detection of vitamin C-mediated H_2_O_2_ generation. In summary the luminescence probe TOB-Eu^3+^ is highly specific for detecting the presence of vitamin C.

### Time-gated luminescence imaging and quantification of extracellular vitamin C uptake in living cells

Before describing luminescence imaging experiments, the influence of TOB-Eu^3+^ on cell proliferation and viability was examined by the MTT assay. As shown in Figure S3, the cell viabilities remained still at above 90% after incubation with up to 100 μM of TOB-Eu^3+^ for 24 h, which indicates that the new probe is biocompatible with low cytotoxicity for the sensing of vitamin C in living cells.

It is well-known that human body cannot synthesize vitamin C because the gene encoding L-gulonolactoneoxidase (GLO), the enzyme required for the last step in ascorbate synthesis, is not functional^25^. Thus, dietary intake of vitamin C becomes vital. The typical human diet contains both vitamin C and DHA, while vitamin C is accumulated in cells by Na^+^-dependent vitamin C transporters (SVCTs), and DHA is absorbed via a Na^+^-independent facilitative glucose transporters (GLUTs) followed by intracellular reduction^26^,^27^,^28^.

**Figure 4:**
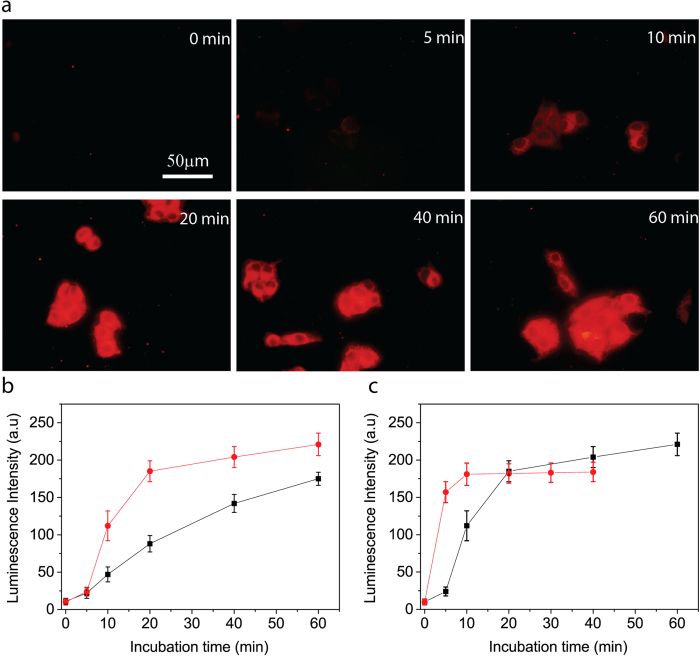
Time-gated luminescence imaging and real-time quantifications of the extracellular vitamin C uptake and intracellular production of vitamin C in living cells. (**a**) Time-gated luminescence microscopy images of HepG2 cells loaded with 1.0 mM vitamin C at different loading times and followed by incubation with 20 μM TOB-Eu^3+^ for 1 h. Scale bar: 50 μm. (**b**) Time-gated luminescence intensities of HepG2 (•) and HeLa (■) cells loaded with 1.0 mM vitamin C for various incubation times and followed by incubation with 20 μM TOB-Eu^3+^ for 1 h. (c) Time-gated luminescence intensity of HepG2 cells loaded with 1.0 mM AA (■) or 1.0 mM DHA (•) for various incubation times and followed by incubation with 20 μM TOB-Eu^3+^.

To evaluate the feasibility of TOB-Eu^3+^ for the luminescent imaging of vitamin C in living cells, the cellular uptake of vitamin C and the endogenous production of vitamin C by intracellular reduction of DHA were monitored using TOB-Eu^3+^ as a probe. For investigating cellular uptake of vitamin C, human HepG2 cells were incubated with an isotonic saline solution containing 1.0 mM vitamin C for various incubation times. Then the vitamin C-loaded cells were incubated with 20 μM TOB-Eu^3+^ probes in the isotonic saline solution containing 0.5 mg/mL solubilizer, cremophor C040 for 1 h under the same condition. The luminescence images of the cells were recorded on a true-colour time-gated luminescence microscope^29^. As shown in Fig. 4, in the beginning of the loading process, cells without internalized vitamin C displayed negligibly weak luminescence. After vitamin C incubation for 10 minutes, the cells showed clearly observable luminescence, and luminescence intensities of cells were gradually increased with the increase of vitamin C loading time. These results demonstrate that the uptake process of vitamin C by HepG2 cells is successfully monitored by using our TOB-Eu^3+^ probe. The amount of the probe per cell as measured by inductively coupled plasma-optical emission spectroscopy (ICP-OES) was ~1.5 × 10^−15^ mol. Taking into account a cell volume of ~4.2 × 10^3^ μm^3^, the intracellular concentration of the probe can be estimated at ~0.36 mM. Compared to the steady-state luminescence image, the background interference from blue autofluorescence of the cells and light scatterings were completely suppressed under time-gated mode, so that a high-contrast and background-free cell image with strongly red luminescence signals was recorded (Figure S4). It is notable that the luminescence signals originated from the reacted probes with the intracellular vitamin C were distributed over the whole cell except the nucleus, which is consistent with the reported fluorescence cell images of vitamin C using hydrophobic phthalocyaninatosilicon fluorophore as a probe^9^. Furthermore even incubated with TOB-Eu^3+^ for 4 h, the HepG2 cells did not show obvious red luminescence in the cells (Figure S5). This result implied that the TOB-Eu^3+^ probe could be stable in the cell and would not be reduced by other naturally existing intracellular reductants.

The TOB-Eu^3+^ probe has been found to be sensitive in quantifying the time-dependent cellular uptake rate of vitamin C by different cell types. Using TOB-Eu^3+^ probe, the cellular uptake of vitamin C by HeLa cells was also monitored (shown in the supplementary Figure S6), and the uptake rate of vitamin C by HeLa cells is slower than that by HepG2 cells (Fig. 4b), which could be due to the higher level express of SVCTs proteins in HepG2 cells^30^.

### Time-gated luminescence imaging and quantification of intracellular production of vitamin C in living cells

Since DHA can be transported into cells and then reduced to vitamin C by intracellular reductants and enzyme systems, our TOB-Eu^3+^ probe has been further verified to monitor the time-dependent production of vitamin C during the cellular uptake process of DHA. In buffer, DHA could not turn on the TOB-Eu^3+^probes, but HepG2 cells loaded by DHA for 5 minutes and incubated with TOB-Eu^3+^ displayed bright red luminescence, and the luminescence intensity increased with the increase of the DHA-loading time (Fig. 4c and supplementary Figure S7). These results illuminate that, after transported into the cells, DHA could be reduced to vitamin C by intracellular reductants and enzyme systems, so that the probes were turned on by the intracellularly produced vitamin C and became strongly luminescent.

The quantification capability and rapid response of the TOB-Eu^3+^ probe enable real-time monitoring of the transportation process of DHA into HepG2 cells or/and the intracellular reduction process of DHA. It is notable that DHA can be rapidly transported into HepG2 cells by GLUTs systems within 10 minutes, while extracellular vitamin C uptake requires approximately 20 minutes by SVCTs system to reach the same intracellular concentration of vitamin C (Fig. 4c). This finding confirms a recent report that the uptake of extracellular vitamin C lasted much longer than the uptake of DHA and its intracellular conversion to vitamin C in EA.hy926 cells (several hours vs. 15 minutes)^31^.

### *In vivo* imaging of vitamin C in laboratory animal

**Figure 5:**
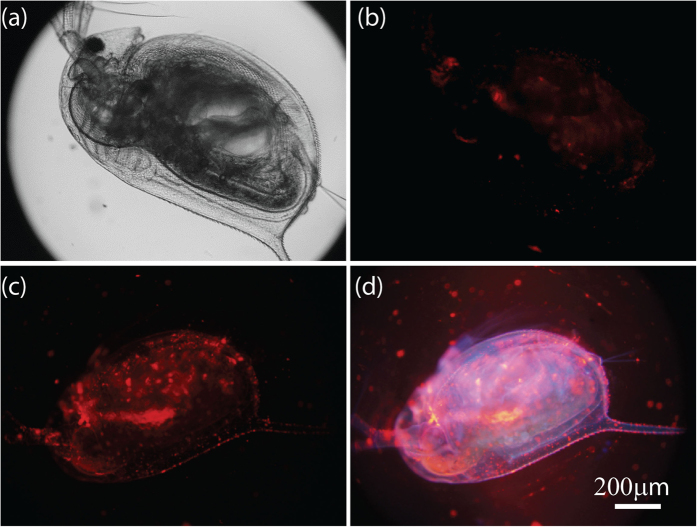
Time-gated *in vivo* imaging of vitamin C in living *Daphnia magna*. Bright-field (**a**) and time-gated luminescence images (**b**) of *Daphnia magna* incubated with 5.0 μM TOB-Eu^3+^ for 1 h. Time-gated (**c**) and steady-state (**d**) luminescence images of *Daphnia magna* loaded with 1.0 mM vitamin C for 40 min and followed by incubation with 5.0 μM TOB-Eu^3+^for 1 h. Scale bar: 200 μm.

A further exploratory effort has been made to evaluate TOB-Eu^3+^ for imaging vitamin C in living *Daphnia magna*, a widely used laboratory animal as an indicator of aquatic ecosystem health and as a model organism in ecotoxicology^32^,^33^. While vitamin C plays an important role in protecting bioorganism against chemical pollutions such as ozone^34^ and organochlorine^35^, it also shows considerable toxicity in the FETAX testing (Frog Embryo Teratogenesis Assay-Xeiiopus)^36^. Largely used in foods, animal feed, pharmaceutical formulations and cosmetic applications, the release of vitamin C into the aquatic environment and its effects on ecotoxicity remain largely unknown. The quantitative *in vivo* imaging of vitamin C in *Daphnia magna* could provide useful insights for understanding its bioaccumulation and effect on aquatic organisms. As shown in Fig. 5, *Daphnia magna*, treated by TOB-Eu^3+^, displayed only weak luminescence, but with vitamin C-loading and TOB-Eu^3+^ incubation, showed strong and clear red luminescence. The head part, midgut and thoracic appendages of *Daphnia magna* exhibited stronger luminescence signals, which might be ascribed to the convenient contact of the probe with vitamin C in these parts. In the absence of time-gated detection as shown in Fig. 5d, *Daphnia magna* emit strongly blue autofluorescence under UV excitation. The red luminescence signals of probes overlapped with the blue autofluorescence of *Daphnia magna* (only a purple color was observed in Fig. 5d), which makes it difficult to unambiguously identify the target. Applying time-gated detection as shown in Fig. 5c, the background autofluorescence is substantially suppressed, so that a highly specific and background-free image of *Daphnia magna* with strong red luminescence signals was recorded.

It should be noted that no immobilization of *Daphnia magna* treated by TOB-Eu^3+^ was observed, and the TOB-Eu^3+^ treatment did not induce a significant change on the heart rate of *Daphnia magna* (~200 beats per minute) during the incubation^37^, which suggests that the probe is biocompatible and low-toxic for *in vivo* imaging of vitamin C in living bodies.

## Discussion

We have successfully developed a responsive luminescence probe, TOB-Eu^3+^, for specific recognition and background-free quantification of vitamin C in living cells and lab animals. Its potential has been comprehensively evaluated by the imaging and quantification of extracellular uptake and intracellular production of vitamin C in cancerous cells and laboratory animals *in vivo*. We presents a useful tool for visualizing the temporal and spatial distribution of vitamin C in cells, biological tissues and living organisms to facilitate the understanding of the roles of vitamin C and DHA in physiological, disease states and ecotoxicology. The rational design of lanthanide-based responsive probes suggests several additional favorable features including high specificity and sensitivity for functional biomolecules, pH-independent luminescence response over the physiological range, and long luminescence lifetime. These features are further enhanced by the high-contrast time-gated luminescence detection of analytes in complex biochemical environments. Therefore, this work further suggests that a library of responsive probes could be designed and synthesized for real-time quantitative imaging of active biomolecules in the living organisms with high specificity and high contrast, immunizing to the background interference from autofluorescence and light scatterings.

## Methods

### Materials and Physical Measurements

BHHBCB was synthesized using previous method^38^. 4-Amino-TEMPO was purchased from TCI. Ascorbic acid, dehydroascorbic acid, dopamine, reduced glutathione, cysteine, succinimide, uric acid and dimethylaminopyridine were purchased from Sigma-Aldrich. Cultured HepG2 cells and HeLa cells were obtained from Dalian Medical University. Cultured *Daphnia magna* were obtained from Professor Jingwen Chen’s group at School of Environmental Science and Technology, Dalian University of Technology. Unless otherwise stated, all chemical materials were purchased from commercial sources and used without further purification.

^1^H NMR spectra were measured on a Bruker Avance spectrometer (400 MHz). Mass spectra were measured on a HP1100 LC/MSD MS spectrometer. Elemental analysis was carried out on a Vario-EL analyser. Time-gated luminescence spectra were measured on a Perkin-Elmer LS 50B luminescence spectrometer with the settings of delay time, 0.2 ms; gate time, 0.4 ms; cycle time, 20 ms; excitation slit, 10 nm; and emission slit, 5 nm. Time-gated luminescence measurements using 96-well microtiter plates as cuvettes were carried out on a Perkin-Elmer Victor 1420 multilabel counter with the settings of delay time, 0.2 ms; window time (counting time), 0.4 ms; cycling time, 1.0 ms; excitation wavelength, 340 nm; and emission wavelengths, 615 nm. All bright-field and luminescence imaging measurements were carried out on a laboratory-use true colour time-gated luminescence microscope^29^. The luminescence quantum yields of TOB-Eu^3+^ and TOHB-Eu^3+^ were measured by an absolute method using an integration sphere on an Edinburgh FLS920 fluorimeter.

### Synthesis of TOB

4-Amino-TEMPO (0.48 mmol, 82.2 mg) was dissolved in anhydrous CH_2_Cl_2_ (5 mL), and then NEt_3_ (0.72 mmol, 0.10 mL), BHHBCB (0.24 mmol, 200 mg) and DMAP (dimethylaminopyridine) (0.024 mmol, 2.9 mg) were added with stirring. The reaction mixture was stirred for 7 days at room temperature in the dark. After solvent was removed, the residue was washed with 50 mL diluted hydrochloric acid (1.0 M), and then dissolved in 150 mL CH_2_Cl_2_. The CH_2_Cl_2_ solution was washed three times with 100 mL water, dried with Na_2_SO_4_ and evaporated to dryness. The desired ligand TOB was obtained as brick-red solid (202.7 mg, 87% yield). ^1^H NMR (400 MHz, DMSO-d_6_): δ (ppm) = 0.95–1.42 (m, br, 16H), 4.20 (s, br, 4H), 6.87–7.91 (m, br, 13H). ES-API MS (m/z): 966.2 ([M-H]^−^, 100%). Elemental analysis calcd.(%) for C_41_H_37_F_14_N_2_O_7_S·1.5H_2_O (TOB·1.5H_2_O): C, 49.50; H, 4.05; N, 2.82. Found (%): C, 49.64; H, 4.29; N, 2.78. HPLC analysis: retention time, 8.01 min (purity, 95.3% integrated intensity); Sinochrom ODS-BP (5 μm) 250 mm × 4.6 mm C18 reverse-phase column; eluent, acetonitrile/H_2_O = 90/10 containing 0.1% trifluoroacetic acid; flow rate, 1.0 mL/min. The elution was monitored at 330 nm.

### Synthesis of TOB-Eu^3+^

TOB (49.7 mg, 0.05 mmol) and europium(III) triflate (16.5 mg, 0.0275 mmol) were dissolved in 5.0 mL CH_3_CN, followed by adding 0.5 mL NaOH (8 mg/mL) dopewise. The reaction mixture was stirred at room temperature for 12 hours. After evaporation, the residue was washed with water and Et_2_O to give the target complex as pale powder. ES-API MS (m/z): 2083.4 ([M-Na]^−^, 100%). Elemental analysis calcd.(%) for C_82_H_70_F_28_N_4_O_14_S_2_NaEu·CH_3_CN·2H_2_O (TOB-Eu^3+^·CH_3_CN·2H_2_O): C, 46.20; H, 3.55; N, 3.21. Found (%): C, 46.23; H, 3.66; N, 3.27. HPLC analysis: retention time, 9.93 min (purity, 95.6% integrated intensity); Sinochrom ODS-BP (5 μm) 250 mm × 4.6 mm C18 reverse-phase column; eluent, acetonitrile/H_2_O = 90/10 containing 0.1% trifluoroacetic acid; flow rate, 1.0 mL/min. The elution was monitored at 330 nm.

### Synthesis of TOHB-Eu^3+^

TOB-Eu^3+^ (43.6 mg, 0.02 mmol) and ascorbic acid (70 mg, 0.4 mmol) were dissolved in 5.0 mL CH_3_CN, and the reaction mixture was stirred at room temperature for 3 hours. After evaporation, the residue was washed with water and CH_3_CN to give the target complex as pale powder. ES-API MS (m/z): 2085.3 ([M-Na]^−^, 100%). Elemental analysis calcd.(%) for C_82_H_72_F_28_N_4_O_14_S_2_NaEu·CH_3_CN·3H_2_O (TOHB-Eu^3+^·CH_3_CN·3H_2_O): C, 45.78; H, 3.70; N, 3.18. Found (%): C, 45.87; H, 3.58; N, 2.91. HPLC analysis: retention time, 6.71 min (purity, 97.2% integrated intensity); Sinochrom ODS-BP (5 μm) 250 mm × 4.6 mm C18 reverse-phase column; eluent, acetonitrile/H_2_O = 90/10 containing 0.1% trifluoroacetic acid; flow rate, 1.0 mL/min. The elution was monitored at 330 nm.

### Reaction of TOB-Eu^3+^ with vitamin C

The reaction of TOB-Eu^3+^ with vitamin C was performed in 1:1 ethanol-0.05 M Tris-HCl buffer of pH 7.5. After 5.0 μM TOB-Eu^3+^ was reacted with different concentrations of vitamin C, respectively, the solutions were 10-fold diluted (final probe concentration, 0.5 μM) with the buffer, and then the excitation and emission spectra of the solutions were measured with time-gated mode.

### Reactions of TOB-Eu^3+^ with different biological reductants

All the reactions were carried out in 1:1 ethanol-0.05 M Tris-HCl buffer of pH 7.5 with the same concentration of TOB-Eu^3+^ (5.0 μM) for 1.5 h at 37 ^o^C. After the reaction, the solutions were 10-fold diluted (final probe concentration, 0.5 μM) with the buffer, and then the luminescence intensities of the solutions were measured with time-gated mode.

### MTT Assay

The cytotoxicity of TOB-Eu^3+^ to HepG2 cells was measured by the MTT assay using the previously reported method^39^. HepG2 cells, cultured in RPMI-1640 medium, supplemented with 10% fetal bovine serum, 1% penicillin and 1% streptomycin, were washed with fresh culture medium, and then incubated with the different concentrations of TOB-Eu^3+^ (0, 25, 50, 75 and 100 μM) for 24 h at 37 °C in a 5% CO_2_/95% air incubator. After the culture medium was removed, the cells were further incubated with the PBS buffer containing 0.5 mg/mL of MTT for 4 h in the incubator. After the supernatants were removed, the cells were dissolved in DMSO, and then the absorbance at 490 nm was measured.

### Luminescence imaging of vitamin C in living Cells

HepG2 cells (or HeLa cells), cultured on a 35 mm glass-bottom culture dish (ϕ20 mm) in RPMI-1640 medium, supplemented with 10% fetal bovine serum, 1% penicillin, and 1% streptomycin, were washed three times with an isotonic saline solution (140 mM NaCl,10 mM glucose and 3.5 mM KCl), and then incubated in the isotonic saline solution containing 1.0 mM vitamin C (or DHA) for various incubation times at 37 ^o^C in a 5% CO_2_/95% air incubator. The vitamin C-loaded cells at different incubation times were washed three times with the isotonic saline solution, and then incubated in the isotonic saline solution containing 20 μM TOB-Eu^3+^ and 0.5 mg/mL cremophor C040 for 1 h under the same condition. The cells were washed five times with the isotonic saline solution, and then subjected to the luminescence imaging measurements on the microscope (excitation filter, 330–380 nm; dichroic mirror, 400 nm; emission filter, >590 nm, no emission filter was used for luminescence imaging in Figure S4). The time-gated luminescence imaging measurements were carried out with the conditions of delay time, 33 μs; gate time, 1.0 ms; lamp pulse width, 80 μs; and exposure time, 2 s. The steady-state luminescence imaging measurements were carried out with exposure time of 4 s.

### Quantifying the intracellular concentration of the probe

The intracellular concentration of the probe was measured by the inductively coupled plasma-optical emission spectrometer (ICP-EOS) assay using the previously reported method^40^. HepG2 cells cultured in a 75 cm^2^ culture flask were washed three times with an isotonic saline solution and then incubated in the isotonic saline solution containing 1.0 mM vitamin C for 1 h at 37 ^o^C in a 5% CO_2_/95% air incubator. The vitamin C-loaded cells washed three times with the isotonic saline solution, and then incubated in the isotonic saline solution containing 20 μM TOB-Eu^3+^ and 0.5 mg/mL cremophor C040 for 1 h under the same condition. The cells were washed three times with PBS and harvested by trypsin treatment. The cell density was determined with a Neubauer improved hemacytometer. The numbered Eu^3+^ complex-deposited cells were suspended in the acidic solution containing 3.7% HCl and 6.5% HNO_3_, and then subjected to the ICP-EOS measurement.

### Luminescence imaging of vitamin C in *Daphnia magna*

*Daphnia magna* were cultured in nonchlorinated tap water that was aerated for 3 days and saturated in dissolved oxygen at 20 ^o^C under cool-white fluorescent light with a 14:10 h light:dark photoperiod. Culture medium was renewed three times a week. *Scenedesmus obliquus* were fed to *Daphnia magna* daily. The new born *Daphnia magna* (age < 48 h) were first exposed to 1.0 mM vitamin C solution in the culture medium for 40 min at 25 ^o^C. After washing three times with culture medium, the vitamin C-loaded *Daphnia magna* were incubated with 5.0 μM TOB-Eu^3+^ in the culture medium containing 1% ethanol for 1 h at 25 ^o^C. The *Daphnia magna* were washed four times with culture medium, and then subjected to the luminescence imaging measurements on the microscope (excitation filter, 330–380 nm; dichroic mirror, 400 nm; no emission filter was used). The time-gated luminescence imaging measurements were carried out with the conditions of delay time, 33 μs; gate time, 1.0 ms; lamp pulse width, 80 μs; and exposure time, 0.5 s. The steady-state luminescence imaging measurements were carried out with exposure time of 1 s.

## Additional Information

**How to cite this article**: Song, B. *et al.* Background-free *in-vivo* Imaging of Vitamin C using Time-gateable Responsive Probe. *Sci. Rep.* **5**, 14194; doi: 10.1038/srep14194 (2015).

## Supplementary Material

Supplementary Information

## Figures and Tables

**Figure 1:**
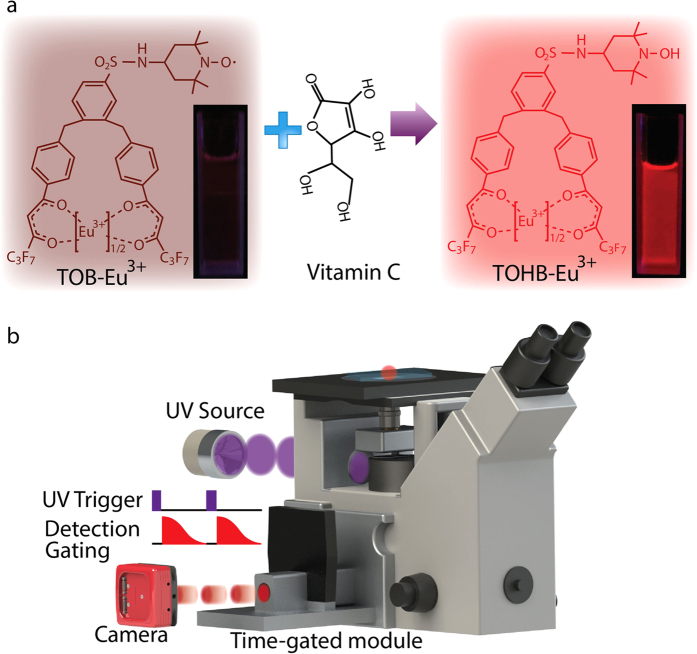
Schematic diagram illustrates the design of a “turn-on” molecular probe specific to vitamin C with long-lived luminescence suitable for time-gated luminescence microscopy (TGLM) system. (**a**) The luminescence probe TOB-Eu^3+^ turns on in its hydroxylamine derivative form of TOHB-Eu^3+^ in the presence of vitamin C (photographs show luminescence colours of the complex solutions under a 365 nm UV lamp). (**b**) The TGLM system employs a rapid-switching flash lamp synchronized to a time-gated optical chopper in front of the camera. The camera is externally shut off during the excitation pulse period, and a short time delay is given to allow the short-lived autofluorescence to vanish before the chopper opens for long-lived luminescence detection in absence of optical backgrounds.

**Table 1:** Luminescence properties of TOB-Eu^3+^ and TOBH-Eu^3+^ in 1:1 ethanol-0.05 M Tris-HCl buffer of pH 7.5.

**Complex**	**λ_ex,max_ (nm)**	**ε_330_ _nm_ (cm^−1^M^−1^)**	**λ_em,max_ (nm)**	**Φ (%)**^1^	**τ (ms)**
TOB-Eu^3+^	329	4.13 × 10^4^	608	7.5	0.18
TOHB-Eu^3+^	332	6.53 × 10^4^	608	73.7	0.38

^1^Quantum yields were measured in 3:1 ethanol-0.05 M Tris-HCl buffer of pH 7.5.
