# Supplementary material for: Background-free in-vivo Imaging of Vitamin C using Time-gateable Responsive Probe
Source: Sci Rep. 2015 Sep 16;5:14194. doi: 10.1038/srep14194 (PMC4570993; doi:10.1038/srep14194)
Supplement: Supplementary Information [file srep14194-s1.pdf]

Supporting information for:

Background-free *in-vivo* Imaging of Vitamin C using Time-gateable Responsive Probe

Bo Song,[1] Zhiqiang Ye,[1] Yajie Yang,[1] Hua Ma,[1] Xianlin Zheng,[2] Dayong Jin,[2] and Jingli Yuan*[1]

[1]State Key Laboratory of Fine Chemicals, School of Chemistry, Dalian University of Technology, Dalian 116024, P. R. China.

[2]Advanced Cytometry Labs, ARC Centre of Excellence for Nanoscale BioPhotonics (CNBP), Macquarie University, NSW 2109,Sydney, Australia.

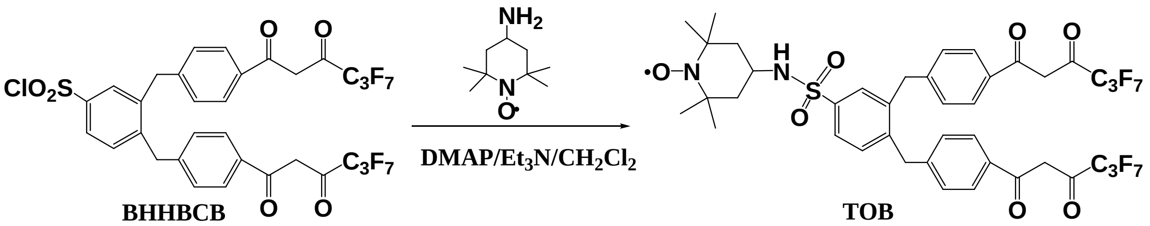

Scheme S1. Reaction pathway for the synthesis of TOB.

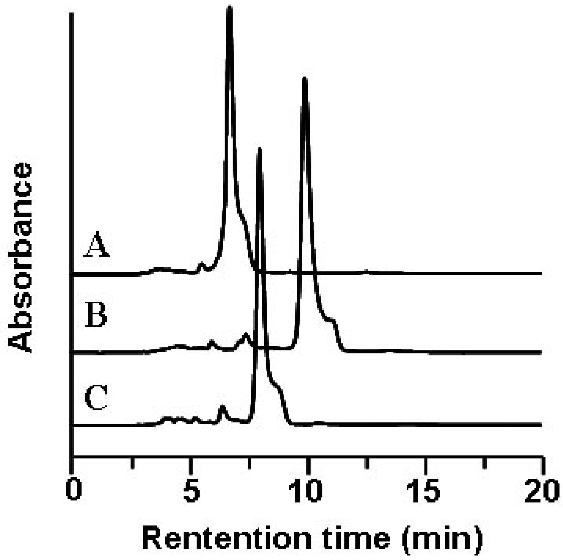

Figure S1. HPLC chromatograms of TOHB-Eu^{3+} (A), TOB-Eu^{3+} (B) and TOB (C).

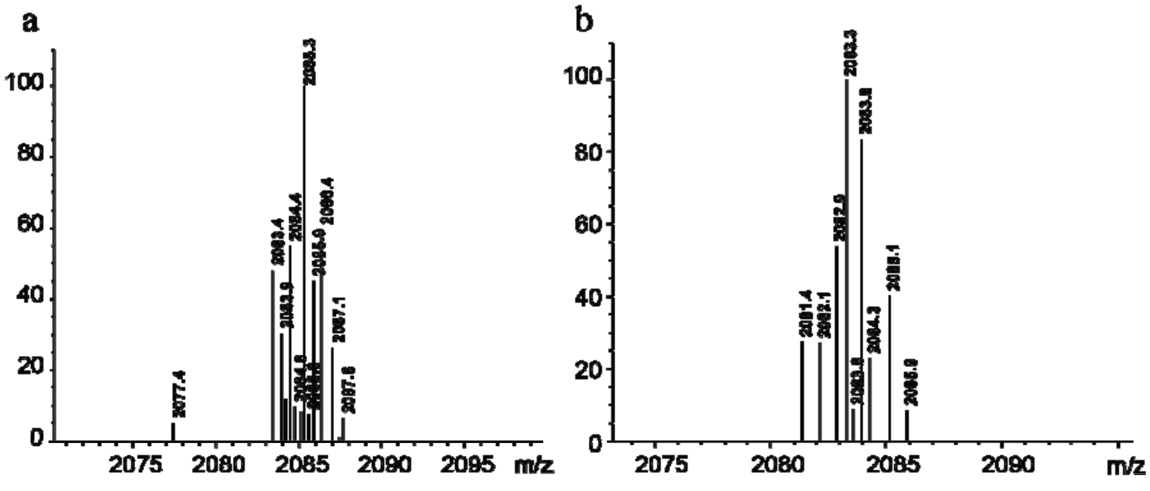

Figure S2. ES-API mass spectra of TOHB-Eu^{3+} (a) and TOB-Eu^{3+} (b).

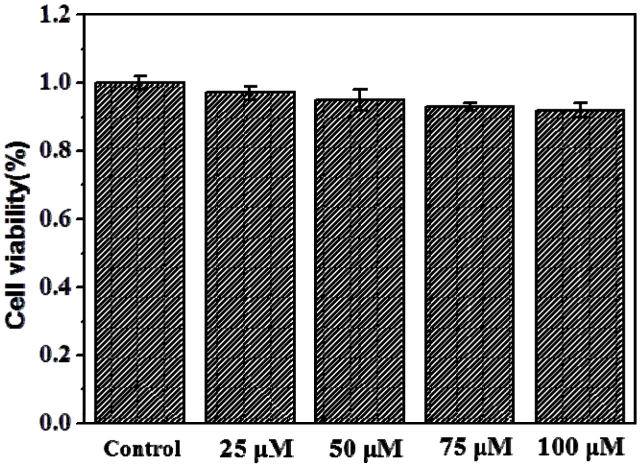

Figure S3. Viabilities of the HepG2 cells after incubated with different concentrations of TOB-Eu^{3+} for 24 h.

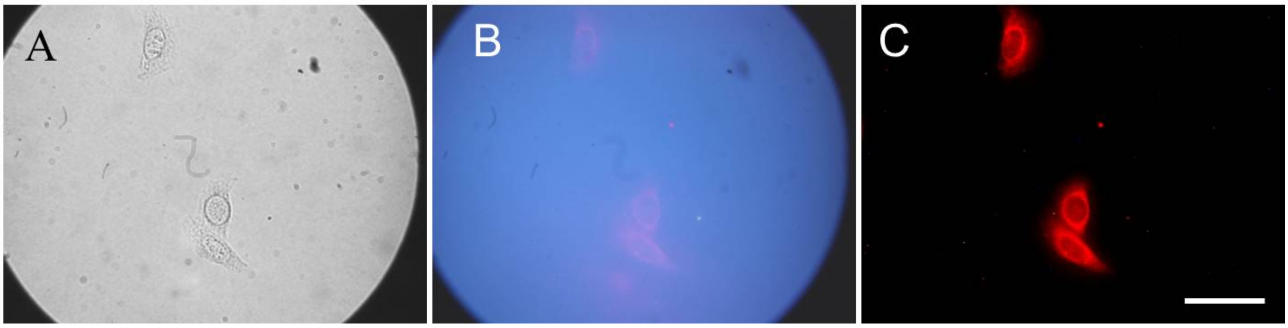

Figure S4. Bright-field (A), steady-state (B) and time-gated luminescence images (C) of HepG2 cells loaded with 1.0 mM vitamin C for 40 min and followed by incubation with 20 µM TOB-Eu^{3+} for 1 h. Scale bar: 50 µm.

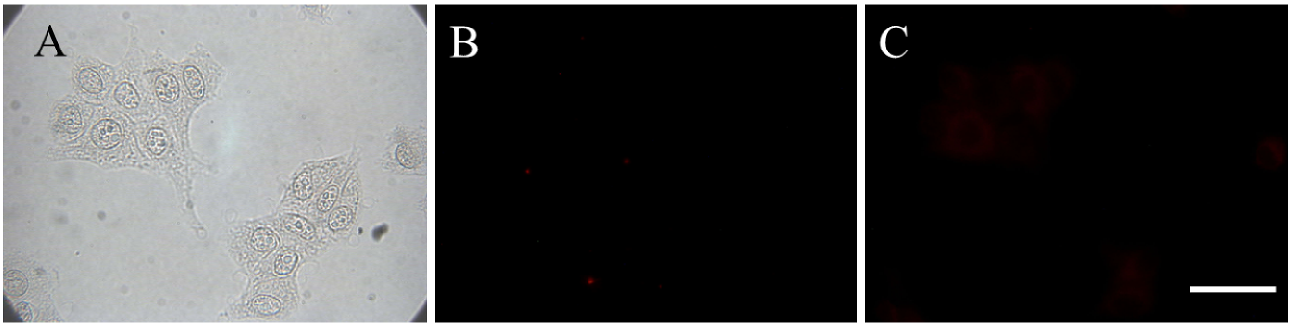

Figure S5. Bright-field (A) and time-gated luminescence images of HepG2 cells incubated with 20 µM TOB-Eu^{3+} for 1 h (B) or 4 h (C). Scale bar: 50 µm.

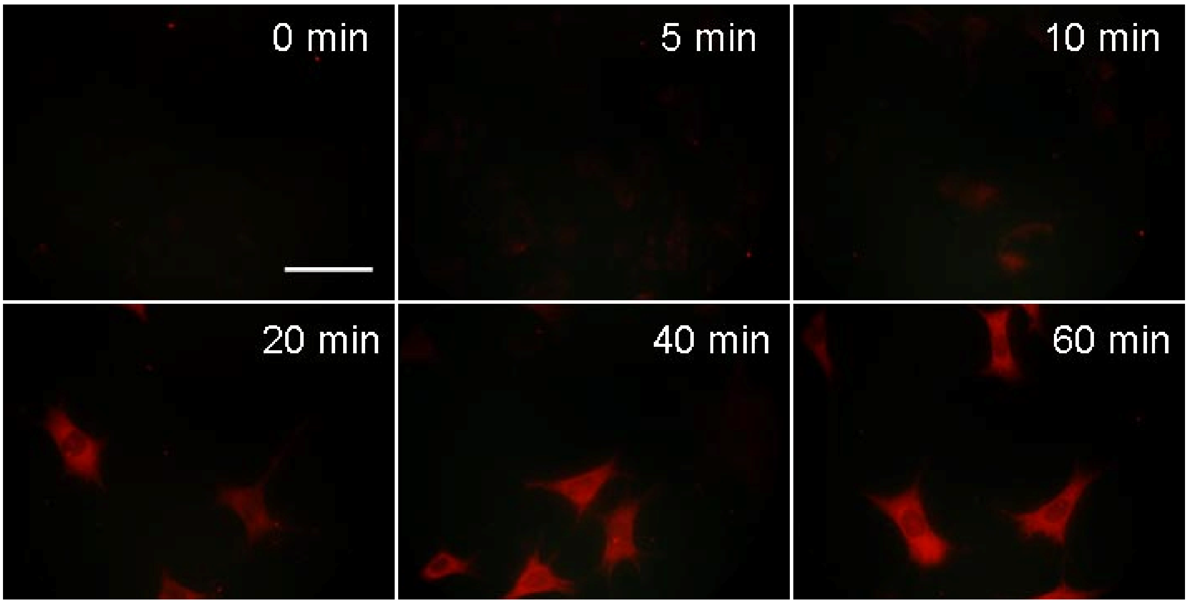

Figure S6. Time-gated luminescence microscopy images of HeLa cells loaded with 1.0 mM AA for various incubation times and followed by incubation with 20 μM TOB-Eu^{3+} for 1 h. Scale bar: 50 μm.

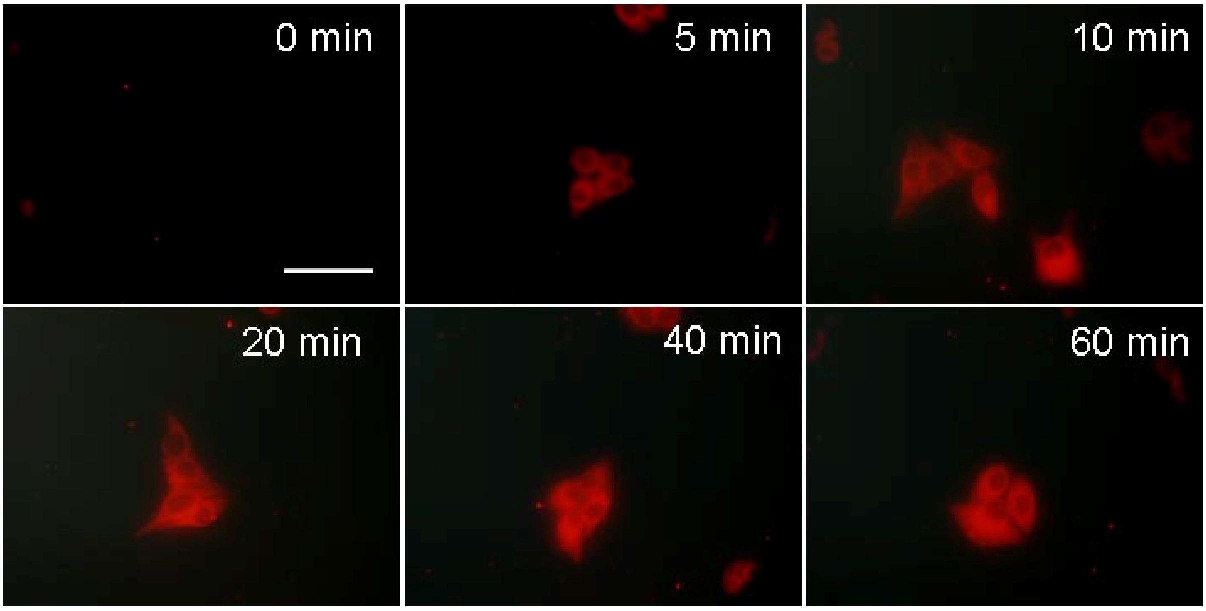

Figure S7. Time-gated luminescence microscopy images of HepG2 cells loaded with 1.0 mM DHA for various incubation times and followed by incubation with 20 μM TOB-$Eu^{3+}$for 1 h. Scale bar: 50 μm.
